# Antibiotics resistance and toxin profiles of *Bacillus cereus*-group isolates from fresh vegetables from German retail markets

**DOI:** 10.1186/s12866-019-1632-2

**Published:** 2019-11-09

**Authors:** Gregor Fiedler, Carmen Schneider, Etinosa O. Igbinosa, Jan Kabisch, Erik Brinks, Biserka Becker, Dominic A. Stoll, Gyu-Sung Cho, Melanie Huch, Charles M. A. P. Franz

**Affiliations:** 1 Department of Microbiology and Biotechnology, Hermann-Weigmann-Straße 1, 24103 Kiel, Germany; 2 Department of Safety and Quality of Fruit and Vegetables, Max Rubner-Institut, Federal Research Institute of Nutrition and Food, Haid-und-Neu-Straße 9, 76131 Karlsruhe, Germany; 3 Present Address: Department of Microbiology, Faculty of Life Sciences, University of Benin, Private Mail Bag 1154, Benin City, 30001 Nigeria

**Keywords:** *Bacillus cereus* sensu lato, Fresh produce, Toxins, Antibiotic resistance, Whole genome sequencing, Food safety

## Abstract

**Background:**

This study aimed to evaluate the safety of raw vegetable products present on the German market regarding toxin-producing *Bacillus cereus* sensu *lato* (*s.l.*) group bacteria.

**Results:**

A total of 147 *B. cereus s.l.* group strains isolated from cucumbers, carrots, herbs, salad leaves and ready-to-eat mixed salad leaves were analyzed. Their toxinogenic potential was assessed by multiplex PCR targeting the hemolysin BL (*hbl*) component D (*hblD*), non-hemolytic enterotoxin (*nhe*) component A (*nheA*), cytotoxin K-2 (*cytK-2*) and the cereulide (*ces*) toxin genes. In addition, a serological test was used to detect Hbl and Nhe toxins. On the basis of PCR and serological results, none of the strains were positive for the cereulide protein/genes, while 91.2, 83.0 and 37.4% were positive for the Hbl, Nhe and CytK toxins or their genes, respectively. Numerous strains produced multiple toxins. Generally, strains showed resistance against the β-lactam antibiotics such as penicillin G and cefotaxim (100%), as well as amoxicillin/clavulanic acid combination and ampicillin (99.3%). Most strains were susceptible to ciprofloxacin (99.3%), chloramphenicol (98.6%), amikacin (98.0%), imipenem (93.9%), erythromycin (91.8%), gentamicin (88.4%), tetracycline (76.2%) and trimethoprim/sulfamethoxazole combination (52.4%). The genomes of eight selected strains were sequenced. The toxin gene profiles detected by PCR and serological test mostly agreed with those from whole-genome sequence data.

**Conclusions:**

Our study showed that *B. cereus s.l.* strains encoding toxin genes occur in products sold on the German market and that these may pose a health risk to the consumer if present at elevated levels. Furthermore, a small percentage of these strains harbor antibiotic resistance genes. The presence of these bacteria in fresh produce should, therefore, be monitored to guarantee their safety.

## Background

The *Bacillus* (*B.*) *cereus* group is of great importance to the food industry as it is associated with food spoilage, resulting in deterioration of food quality, as well as of food safety, affecting public health and the economy. Bacterial toxins ranked second in 2016 as a group of causative agents for foodborne outbreaks in Europe [1]. *Bacillus cereus* group species are readily isolated from food crops and food plants [2–4]. Significant numbers of bacteria belonging to the *B. cereus* group have been shown to occur in dried spices and herbs, but also in fresh herbs and vegetables (carrots, leaf lettuce, cucumbers and mixed salad leaves) in Germany [2, 5, 6]. The presence of both vegetative cells and spores in food commodities has been reported and their role in food safety and food spoilage is well known [7]. In 2016, the European Union (EU) reported 413 foodborne outbreaks being caused by *Bacillus* toxins affecting 6657 people. Of these 352 people were hospitalized without terminal outcome [7]. However, certain severe cases of foodborne illness by *B. cereus* leading to mortality or organ failure have sporadically been reported in several countries [8–10].

*Bacillus cereus* belongs to the *B. cereus* group of bacteria, also known as *B. cereus* sensu *lato* (*s.l.*), which consists of eight genetically closely related species that include *B. anthracis*, *B. cereus* sensu stricto (*s.s.*), *B. cytotoxicus*, *B. mycoides*, *B. pseudomycoides*, *B. thuringiensis*, *B*. *toyonensis* and *B. weihenstephanensis* [11–14]. The taxonomic relationships between members of the *B. cereus* group are controversially debated and identification based on 16S rRNA gene sequencing fails due to high nucleotide conservation in the sequence of this gene among species of this group [14]. The species occurring within the *B. cereus s.l.* have so far been distinguished on the basis of morphological, physiological and/or virulence characteristics, the latter which includes characterization of toxin genes that are often located on extrachromosomal genetic elements (plasmids), as well as multilocus sequence analysis and phylogenomics [14].

*Bacillus cereus s.s.* is an opportunistic pathogen capable of causing a range of diseases [15], most prominently foodborne disease due to the production of enterotoxins (diarrheal toxin) or a non-ribosomal peptide synthetase (NRPS) toxin (emetic or cereulide toxin) [16]. The diarrheal toxins hemolysin BL (Hbl) non-hemolytic enterotoxin (Nhe) and cytotoxin K (CytK) [17, 18] have been linked to the diarrheal type of *B. cereus* food poisoning, which is characterized by abdominal pain and watery diarrhea. The Hbl and Nhe enterotoxins are three-component toxins consisting of the subunits HblC, HblD and HblA for Hbl toxin and the subunits NheA, NheB and NheC for the Nhe toxin. The virulence of emetic strains is attributed to the production of a heat-stable cereulide, synthesized by a non-ribosomal peptide synthetase encoded by *ces* genes [16].

The growing challenge of the emergence and spread of antibiotic resistance has been the subject of several surveys [19] and has been reported by the World Health Organizations as one of the major health challenges of the twenty-first century [20, 21]. *Bacillus cereus s.s.* is typically resistant to penicillin and other β-lactam antibiotics [22] and can furthermore acquire resistance to commonly used antibiotics such as ciprofloxacin, cloxacillin, erythromycin, tetracycline and streptomycin [22, 23]. Foodborne illness associated with *B. cereus* group strains seldomly need to be treated with antibiotics. However, it has not been well investigated to which degree *B. cereus* group-strains can serve as a source of transferable antibiotic resistance genes in the food chain.

In this study, bacterial strains from fresh vegetables on the German market were identified as belonging to the *B. cereus s.l.* group by 16S rRNA gene sequencing, and their potential for toxin production was determined. This was done to determine the toxinogenic potential of these organisms in fresh produce. For this, a multiplex PCR based on the toxin genes *hblDA, nheAB, cytk* and *ces* as target genes was used. The multiplex PCR was done in independent replicates in two different laboratories with different polymerase enzymes to determine the reproducibility of the methods. Apart from molecular analysis, the expressed bacterial toxins NheB and HblC (HblL_2_) were also detected using a commercially available immunological assay. In addition, the phenotypic antibiotic resistances of strains were determined and the antibiotic resistance genes of selected resistant isolates were characterized by whole genome sequencing. Furthermore, whole genome sequence data were used to investigate differences in toxin gene typing by either PCR or serological testing. Therefore, the study aimed to provide an indication on the risk of *B. cereus s.l.* strains in vegetables on the German market from both a food safety and antibiotic resistance point of view.

## Results

### Phenotypic and genotypic characterization

*B. cereus*-group isolates were characterized according to their typical colony characteristics and to their growth behavior at different temperatures. Only very few (7 of 147 strains, 4.8%) were able to grow at 4 °C, while a moderate percentage of strains were able to grow at 7 °C (45 out of 147, 30.6%) and at 50 °C (40 out of 147, 27.2%). Nearly all strains showed β-hemolysis, except 2 out of 147 strains (1.4%) which were not hemolytic (γ-hemolytic). The 16S rRNA genes of all strains were sequenced and the strains all clustered closely together with the type strains of the *B. cereus s.l.* group, namely *B. cereus s.s., B. toyonensis, B. weihenstephanensis, B. anthracis, B. mycoides*, *pseudomycoides* and *B. thuringiensis* at *r* = 98.8% (Additional file 1: Figure S1). The type strains of *B. cytotoxicus* clustered at *r* = 97.6% with the above mentioned strains. On the other hand, the 16S rRNA gene sequences clustered apart from the type strain of *B. subtilis,* showing that all isolates in this study should be considered as belonging to the *B. cereus s.l.* group.

**Fig. 1:**
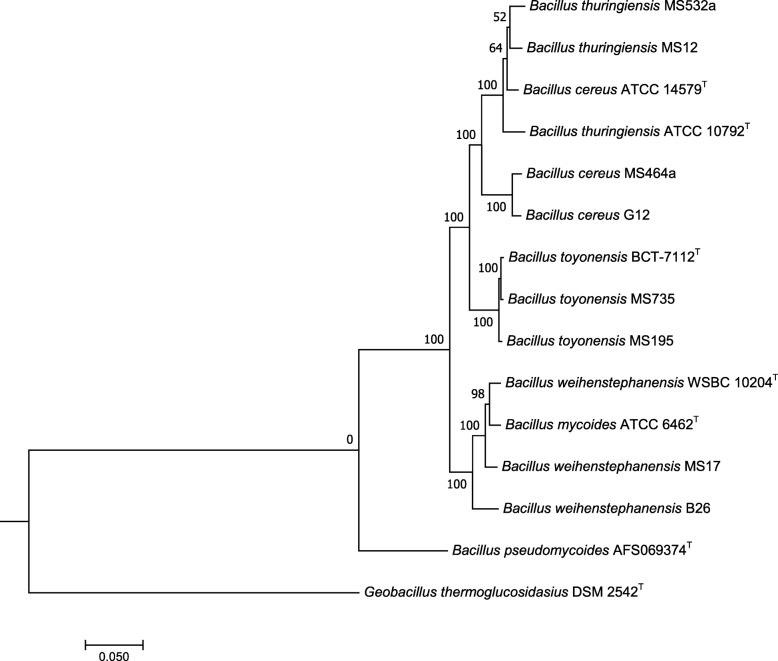
FastTree phylogenetic comparison with whole genome data. A homologous group filtering and a group alignment were performed by PATRIC pipeline [24] and an estimated phylogenetic tree from concatenated alignment sequences was calculated within the type strains *B. cereus* ATCC 14579^T^ (DSM 31^T^), *B. thuringiensis* ATCC 10792^T^ (DSM 2046^T^), *B. toyonensis* BCT-7112^T^ (CECT 876^T^), *B. weihenstephanensis* WSBC 10204^T^ (DSM 11821^T^), *B. mycoides* ATCC 6462^T^ (DSM 2048^T^) and *Bacillus pseudomycoides* AFS069374^T^ using a FastTree method [25]. *Geobacillus thermoglucosidasius* DSM 2542^T^ was used as outgroup strain

Eight strains of the *B. cereus s.l.* group isolates were selected as they either showed resistance towards one or more antibiotics, or because they represented particular enterotoxin types. The genome sequences of the eight selected strains were also used for genome-based phylogenetic comparison and the strains were clustered into one of three groups (Fig. 1). Four strains (532a, MS12, MS464a and G12) clustered together with the *B. cereus* ATCC 14579^T^ (DSM 31^T^) and the *B. thuringiensis* ATCC 10792^T^ (DSM 2046^T^) type strains. Two strains (MS735 and MS195) clustered together with the *B. toyonensis* BCT-7112^T^ type strain, while two (MS17 and B26) clustered with the *B. mycoides* ATCC 6462^T^ (DSM 2048^T^) and the *B. weihenstephanensis* WSBC 10204^T^ (DSM 11821^T^) type strains (Fig. 1).

### Toxin gene profiles

The toxin gene profiles established in this study using multiplex PCR at two research laboratories are shown in Table 1. While the *hblDA* genes could be determined in 134 of the 147 (91.2%) isolates in laboratory 1, these genes could be shown to be present in only 128 of the 147 isolates (87.1%) in laboratory 2. The *nheAB* genes were present in approximately equal numbers of strains, i.e. in 108 vs. 107 of the 147 strains (72.8% vs. 73.5%) in laboratories 1 and 2, respectively. The *cytK-2* gene was detected in both departments in 55 of the 147 strains (37.4%), while none of the strains could be shown to contain the *ces* gene.

**Table 1:** Incidences of toxin genes *hbl*, *nhe*, *cytK-2* and *ces* detection and of toxins Nhe and Hbl expression

Department/laboratory	Presence of toxin gene detected in multiplex PCR				Toxin gene profile (classification according to Ehling-Schulz et al. [16])					Duopath® test for toxin production	
	*hblD*	*nheA*	*cytK-2*	*ces*	*hbl*+/*nhe*+/ *cytK*+/*ces*- (A)	*hbl*+/*nhe*+/ *cyt*K−/*ces*- (C)	*hbl*+/*nhe−*/ *cytK+*/*ces*- -	*hbl−/nhe*+/ *cyt*K−/*ces*- (F)	*hbl−/nhe-cytK*+/*ces*- (G)	Nhe	Hbl
Microbiology and Biotechnology (Kiel) Laboratory 1	134/147 (91.2%)	108/147 (73.5%)	55/147 (37.4%)	0/147 (0%)	16/147 (10.9%)	79/147 (53.7%)	39/147 (26.5%)	13/147 (8.8%)	0/147 (0%)	122/147 (83.0%)	93/147 (63.3%)
Safety and Quality of Fruit and Vegetables (Karlsruhe) Laboratory 2	128/147 (87.1%)	107/147 (72.8%)	55/147 (37.4%)	0/147 (0%)	15/147 (10.2%)	78/147 (53.1%)	35/147 (23.8%)	14/147 (9.5%)	5/147 (3.4%)		
Combined result of multiplex PCR and Duopath® test for toxin production											
Microbiology and Biotechnology (Kiel) Laboratory 1					55/147 (37.4%)	79/147 (53.7%)	0/147 (0%)	13/147 (8.8%)	0/147 (0%)		
Safety and Quality of Fruit and Vegetables (Karlsruhe) Laboratory 2					55/147 (37.4%)	79/147 (53.7%)	0/147 (0%)	13/147 (8.8%)	0/147 (0%)		

According to the toxin gene profiles of Ehling-Schulz et al. [16] the majority of strains (79 of 147; 53.7%) encoded the *nheAB* and *hblDA* genes and thus belongs to the toxin gene profile C (Table 1) in this study. In addition, about 25% strains were found to encode the *hblDA* and *cytK* genes which was not defined as a toxin gene profile by Ehling-Schulz et al. [16]. The other toxin gene profiles F (only the *nheAB* toxin genes) and G (only *cytk* toxin genes) were determined to occur at ca. 9% and ca. 3% in this study (Table 1).

The Duopath® test for detection of toxin production showed a positive result for the Nhe toxin in 122/147 of the strains (83.0%), while the Hbl toxin was detected in 93 (63.3%) of the strains. When the toxin production data as determined with the Duopath® test were considered together with the multiplex PCR data, the number of strains belonging to toxin gene profile A (*hbl*+, *nhe*+, *cyt*K+, *ces*-) increased from approx. 10 to 37.4% of strains, while the number of strains belonging to toxin profile C and F stayed the same at 53% and ca. 9%, respectively (Table 1). The Duopath® test was able to increase the number of Nhe-positive strains assessed on the basis on the presence of the *nheAB* gene, as in the multiplex PCR approx. 73% of strains possessed the *nheAB* gene, while in the Duopath® test 83% of strains produced Nhe. A different situation existed for assessment on the incidence of the Hbl toxin, as the multiplex PCR showed 91.2% (laboratory 1) or 87.1% (laboratory 2) of strains to possess the *hblDA* gene, while toxin production assessment with the Duopath® test showed only 63.3% of strains to be positive (Table 1).

### Antibiotic resistance

**Fig. 2:**
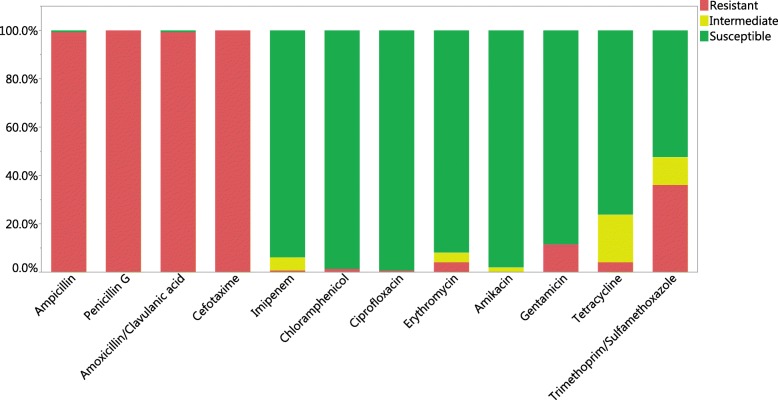
Antibiotic resistance profile of *Bacillus* strains based on inhibition zone diameter (mm); (red) resistance, (yellow) intermediate, (green) susceptible

The *B. cereus*-group strains were generally resistant to the β-lactam antibiotics penicillin G (PEN) and cefotaxime (CTX) (100%), as well as ampicillin (AMP) and amoxicillin/clavulanic acid combination (AMC) (99.3%) (Fig. 2). On the other hand, these strains were generally susceptible to ciprofloxacin (CIP) (99.3%), chloramphenicol (CHL) (98.6%), amikacin (AMK) (98.0%), imipenem (IPM) (93.9%), erythromycin (ERY) (91.8%), gentamicin (GEN) (88.4%) and tetracycline (TET) (76.2%), while 52.4% of strains were susceptible to trimethoprim-sulfamethoxazol (SXT) (Fig. 2).

Conversely, only few strains were resistant to the antibiotics CHL (1.4%), CIP and IPM (0.7%), ERY (4.1%), GEN (11.6%) and TET (4.1%), while more (36.1%) were resistant to SXT, as based on the EUCAST breakpoints (Fig. 2). Intermediate susceptible strains were also determined for AMK (2.0%), ERY (4.1%), IMP (5.4%), TET (19.7%) and SXT (11.6%).

**Fig. 3:**
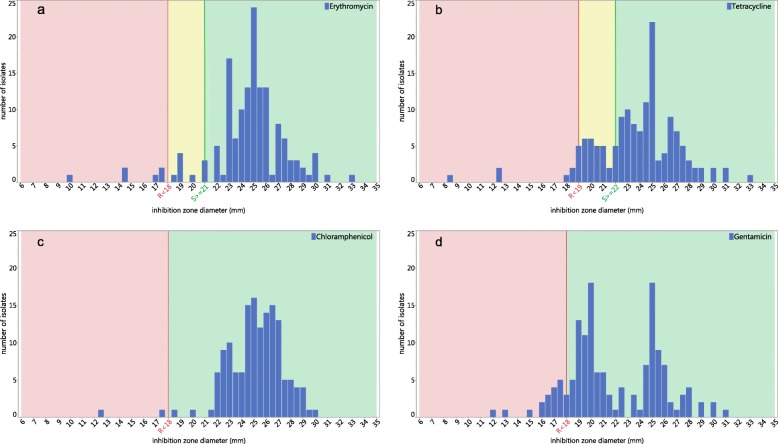
Distribution of inhibition zone diameter in the antibiotic disc diffusion test. Each average value was obtained from triplicate test. **a** erythromycin, **b** tetracycline, **c** chloramphenicol, **d** gentamicin. Green indicates susceptible, yellow intermediate and red resistant to antibiotics

The inhibition zone ranges (in mm diameter) of the different antibiotics at their respective concentrations used in this study were investigated more closely. Such inhibition zones grouping the isolates together (Fig. 3 a-d) ranged from 17 to 33 mm in the case of ERY, 18 to 33 mm for TET, 21.5 to 30 mm for CHL and 15 to 31 mm for GEN. Isolates exhibiting smaller sized inhibition zones outside this range, which were also considered to be resistant according to the EUCAST guidelines [26] (for *S. aureus*), were interesting candidates for investigating the genetic basis of the antibiotic resistance by whole genome sequencing.

### Whole genome sequencing

The genomes of selected antibiotic-resistant strains were sequenced (Table 2) and genome sizes ranged from 5.17 to 6.35 Mbp, while the mol% G + C content ranged from 34.70 to 35.31. Liu et al. [14] reported that within a collection of 224 *B. cereus s.l.* strains, the genome sizes ranged from 4.09 to 7.09 Mbp with an average 5.77 Mbp, which agreed well with the genome sizes determined in our study. Furthermore, the mol% G + C values of these genomes were reported to range from 34.5 to 36.7% [14]. Again, the data from the genome sequences obtained for the strains in this study agree well, as they fit within this range. Sequencing the genomes of the selected isolates confirmed the results of the multiplex PCR (in combination with serological testing) for *B. cereus s.l.* enterotoxin genes, as the genes detected on the genomes with automated annotation were the same enterotoxin profile as detected by combined multiplex PCR and enterotoxin serological testing results (Table 2).

**Table 2:** Characteristics of whole genome datasets of selective *B. cereus s.l.* strains

Characteristic	*Bacillus cereus s.l.* isolate							
	B26	G12	MS12	MS17	MS195	MS464a	MS532a	MS735
No. of contigs	67	64	368	48	79	41	75	77
Largest contig	524,166	855,854	262,491	733,489	594,878	746,015	1,014,035	657,340
N_50_	228,834	193,941	44,422	251,250	146,479	249,433	154,418	201,858
GC-content (mol%)	35.27	35.08	34.73	35.31	34.90	35.27	34.70	34.89
Total length (bp)	5,439,456	5,686,136	6,345,988	5,472,616	6,018,360	5,170,399	6,113,939	5,964,825
MLST (*B. cereus*)	ST-352	unknown	ST-15	unknown	ST-278	ST-163	unknown	ST-72
Species (KmerFinder)	*Bacillus weihenstephanensis*	*Bacillus cereus*	*Bacillus thuringiensis*	*Bacillus weihenstephanensis*	*Bacillus toyonensis*	*Bacillus cereus*	*Bacillus thuringiensis*	*Bacillus toyonensis*
Antibiotic resistance phenotype^A^	AMP, CTX, AMG, PEN, SXT, GM	AMP, CTX, AMG, PEN, SXT	AMP, CTX, AMG, PEN	AMP, CTX, AMG, PEN, SXT	AMP, CTX, AMG, PEN, ERY,	AMP, CTX, AMG, PEN, SXT, GEN	AMP, CTX, AMG, PEN, TET, GEN, ERY, CHL	AMP, CTX, AMG, PEN, ERY,
Enterotoxin PCR	*nhe+, hbl+, cytK, ces*^*−*^	*nhe+, hbl+, cytK*^*+*^*, ces*^*−*^	*nhe-, hbl+, cytK*^*+*^*, ces*^*−*^	*nhe+, hbl*^*−*^*, cytK*^*−*^*, ces*^*−*^	*nhe+, hbl+, cytK, ces*^*−*^	*nhe+, hbl*^*−*^*, cytK*^*−*^*, ces*^*−*^	*nhe+, hbl+, cytK*^*+*^*, ces*^*−*^	*nhe+, hbl+, cytK*^*−*^*, ces*^*−*^
Enterotoxin serology	Nhe+, Hbl^−^	Nhe+, Hbl^+^	Nhe+, Hbl^+^	Nhe+, Hbl^−^	Nhe+, Hbl^+^	Nhe+, Hbl^−^	Nhe+, Hbl^+^	Nhe+, Hbl^+^
Enterotoxin genes identified on genome	*nhe* and *hbl* genes	*nhe*, *hbl* and *cytK* genes	*nhe*, *hbl* and *cytK* genes	*nhe* and *hbl* genes	*nhe* and *hbl* genes	*nhe* and *hbl* genes	*nhe*, *hbl* and *cytK* genes	*nhe* and *hbl* genes

Species identification was carried out with KmerFinder (PATRIC) which examined the number of co-occurring k-mers (substrings of k nucleotides in genome sequence data) and MLST was done by PATRIC to assign the strains to clonal lineages. Abbreviations^A^: amikacin (AMK), ampicillin (AMP), amoxicillin/clavulanic acid (AMG), cefotaxime (CTX), chloramphenicol (CHL), ciprofloxacin (CIP), erythromycin (ERY), gentamicin (GEN), imipenem (IPM), penicillin G (PEN), tetracycline (TET) and trimethoprim-sulfamethoxazole (SXT)

**Fig. 4:**
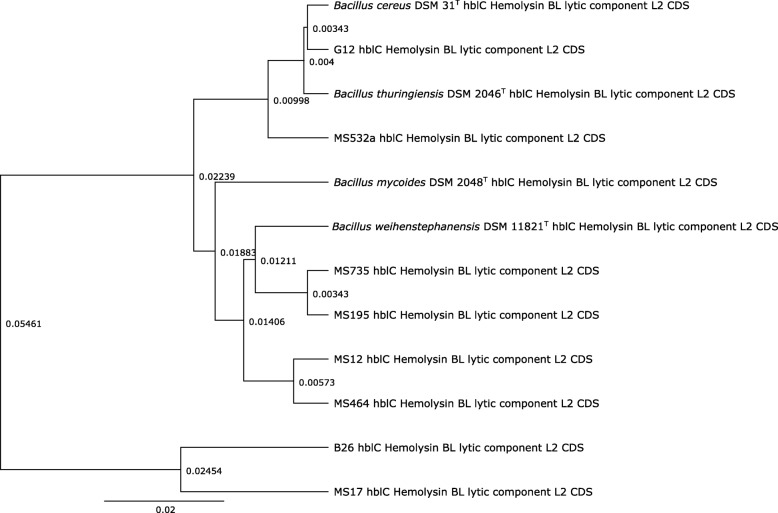
The amino acid sequences of the hblC gene of *Bacillus cereus s.l.* strains were clustered in this study. Most of strains reacted Hbl-positive with the Duopath test but two strains (B26 and MS17) were Hbl-negative. The phylogenetic analysis was carried out using the Jukes-Cantor model for genetic distance and the unweighted pair-group method using arithmetic averages (UPGMA) algorithm of the Geneious® program. The branch length and branch support values indicate the units of substitutions per site of the sequence alignment

Concerning the results of the enterotoxin gene analyses by multiplex PCR and serological testing, some interesting discrepancies occurred in the results. For example, in strain MS12 no PCR product could be obtained in the *nheAB* PCR, yet a signal was obtained using the Duopath® test and genome sequencing showed the genes to be present. Clearly, this must have depended on polymorphisms in the *nheAB* gene nucleotide sequence, that resulted in no or insufficient primer binding. Indeed, the *nheAB* forward primer (NA2-F) showed a T/C mismatch for strain MS12, which was present in the PCR primer target sequence of only this strain (Additional file 2: Figure S2a). Furthermore, for strains B26 and MS17, the Hbl toxin results were somewhat confusing. Strain B26 showed a PCR signal in the multiplex PCR for the *hblDA* genes, while strain MS17 did not. The genome sequence of both strains B26 and MS17 showed the Hbl operon to be present. Thus, the lack of *hblDA* signal in PCR for strain MS17 can be explained by a G/A primer mismatch for primer hbl F (HD2 F) (Additional file 2: Figure S2b). Moreover, in the serological assay, the Hbl toxin (HblC subunit) could not be detected for both strains B26 and MS17, indicating that possibly the amino acid sequence differences in this gene *hblC* in these two strains did not allow the antibody to react with the toxin. Performing a phylogenetic tree with the amino acid sequence of the HblC proteins showed a clear separation of B26 and MS17 from the other six strains (Fig. 4). Alternatively, it is known that the detection limit of the Duopath® test kit is 20 ng/ml and possibly the strain did not produce sufficient toxin to show a serological reaction.

The genome sequencing showed that all sequenced strains contained a gene encoding a fosfomycin resistance protein FosB, a broad specificity multidrug efflux pump YkkC and a gene encoding a putative streptogramin O-acetyltransferase involved in streptogramin A resistance (Additional file 3: Table S1). Genes encoding β-lactam antibiotic resistances present on the genome generally included genes for class A β-lactamases (EC 3.5.2.6) and class B (subclass B1) β-lactamases (Additional file 3: Table S1).

Of the eight sequenced strains, three were resistant to erythromycin. These three strains possessed each two or more genes associated with macrolide resistance (macrolide 2′-phosphotransferase of the Mph(B) family, putative macrolide 2′-phosphotransferase and ABC-F type ribosomal protection protein Lsa(B)). However, other strains were susceptible to erythromycin, even though the same genes were present (Additional file 3: Table S1). Strain MS532a possessed a *tet*(45) tetracycline resistance gene, encoding an efflux pump (Additional file 3: Table S1). This strain showed a tetracycline resistant phenotype and displayed an inhibition zone of only 8.5 mm in the disc diffusion test. Six of the eight sequenced strains contained a chloramphenicol *O*-acetyltransferase gene (Additional file 3: Table S1), but of these only one (MS532a) displayed a chloramphenicol-resistant phenotype. All strains contained an aminoglycoside 6-nucleotidyltransferase gene, as well as a putative gene with weak similarity to an aminoglycoside N(3)-acetyltransferase. However, none of the strains showed resistance to amikacin and only three strains displayed gentamicin resistance.

## Discussion

The 16S rRNA gene sequence showed that bacteria in this study isolated from *B. cereus* selective agar all belonged to the *B. cereus s.l.* group. The intention of this study, however, was not to accurately distinguish between the different members of the *B. cereus s.l.* group, but only to confirm that the isolates were members of this group. To address the importance of these bacteria in the context of food safety, it was more important to determine whether they possessed gene(s) for toxin production. Nevertheless, our phylogenomic assessment of the eight sequenced strains showed that strains from vegetables in this study consisted of a mix of species, including *B. toyonensis*, *B. cereus/thuringiensis* and *B. mycoides/weihenstephanensis* (Fig. 1).

Our multiplex PCR results showed that a large proportion (91.2%) of the *B. cereus*-group strains from fresh produce possessed the *hbl* gene, while 73.5% of the strains possessed the *nhe* and 37.4% possessed the *cytK* genes. This was slightly lower than reported for *Bacillus cereus s.l.* group strains isolated from dried spices and herbs in Germany [5]. In that investigation 94.9% of strains possessed the *hbl* gene, while the *nhe* gene and the *cytK* genes were present in 96.6 and 50.8% of strains, respectively [5]. Other reports have also shown that the *nhe* gene sequence is detected at high frequency [27, 28]. Park et al. [29] detected the *nhe*, *hbl* and *cytK* genes in 92, 93 and 55% of *B. cereus* strains from cereals, respectively. The lower prevalence of *cytK* genes observed in this study was also in accordance with other studies [5, 29, 30]. *Bacillus cereus* strains possessing the *cyt*K gene have a high pathogenic potential, as the *cytK* gene was frequently detected in strains associated with diarrhea and food poisoning [29, 31]. The results for multiplex PCR from laboratory 1 and laboratory 2 differed only slightly, which may be explained by the small variations in PCR protocol, PCR chemicals (including type of polymerase used) as well as PCR equipment.

Our combined multiplex PCR and Duopath® tests suggested that half of the strains (53.7%) were positive for both Hbl and Nhe, while more than a third (37.4%) were positive for all three toxins Nhe, Hbl and CytK. In the study of Frentzel et al. [5] 49.2% of strains possessed all the three *hbl*, *nhe* and *cytK* genes, while 45.8% possessed the genes for *hbl* and *nhe*, only. In the study of Frentzel et al. [5] a single strain was found to possess the *ces* gene for cereulide production, while this gene could not be found among any of the strains in this study.

These results suggested that toxinogenic *B. cereus s.l.* group organisms occur in spices, herbs and fresh produce in Germany and that these commonly contain genes for diarrheal toxin production, while the occurrence of strains producing emetic toxin is rather low. Our study confirmed previous reports that the *nhe* and the *hbl* toxin genes were most prevalent, and the *cytK* enterotoxin genes occurred at a lower incidence than these two [5, 29]. Böhm et al. [32] showed a strictly vertical inheritance for the *nhe* gene, which may explain why this gene is more prevalently occurring in *B. cereus s.l.* strains, while there was ample evidence for duplication of *hbl*, horizontal transfer of *hbl* and *cyt*K, as well as frequent deletion of both toxins. The latter may explain the lower occurrence of the cytK toxin.

In our previous investigation on the microbiological quality and safety of fresh produce from the German market, we reported that *B. cereus s.l.* counts in fresh produce such as cucumbers, carrots, herbs, leaf lettuce and mixed salad leaves obtained directly from retail close to the end of the use by date are quite low, with mean counts ranging from 1.26 × 10^1^ to 5.1 × 10^1^ cfu/g [2]. Becker et al. [6] and Frentzel et al. [5] reported *B. cereus s.l*. counts in salads and herbs, and in dried spices and herbs from the German market to range from ca. 10^2^ to 10^4^ cfu/g and < 8.0 × 10^1^ to 1.6 × 10^3^ cfu/g, respectively. These findings are furthermore in agreement with reports of Banerjee and Sarkar [33], Kneifel and Berger [34] and Sagoo et al. [35], who reported presence of only low (less than 1 × 10^4^ cfu/g) levels of *B. cereus-*group strains in condiment samples. *Bacillus cereus* diarrheal toxins are formed in the human gut after consumption of cells or spores in quantities of usually more than 1 × 10^5^ cfu/g [1, 5, 36]. These data, therefore, indicate that *B. cereus-*group strains containing mostly enterotoxin genes are present in dried herbs and spices, as well as fresh produce on the German retail market and that they do pose an inherent risk to consumers. However, usually these occur at too low numbers to cause intoxication.

The increased occurrence of multiple antibiotic resistance is currently a concern for food processors and public health officials. As fresh produce can be eaten without further heat treatment, there is a concern that antibiotic-resistant bacteria may survive gastrointestinal passage and may complicate the treatment in the very young or old consumers or persons with lowered immune function after infection. For this reason, we also investigated the incidence of antibiotic resistances in the *B. cereus s.l*. isolates obtained from fresh produce. *Bacillus cereus* group strains are known to be typically resistant towards β-lactam antibiotics as a result of production of β-lactamase enzymes [29, 37–39]. This was confirmed in this study, which showed that strains were generally resistant to penicillin G, ampicillin, cefotaxime and the combination of amoxicillin with the clavulanic acid as β-lactamase inhibitor. Similar to the results of other studies [29, 38, 39], these bacteria were generally susceptible towards other antibiotics, including tetracycline, erythromycin, chloramphenicol, gentamicin, ciprofloxacin and imipenem. A few strains did, however, show reduced susceptibility to gentamicin, tetracycline and erythromycin (Figs. 2 and 3). Resistances to tetracycline and erythromycin in these bacteria have previously been noted to occur in the United States and Europe [38].

The genomes of three erythromycin-resistant and five erythromycin-susceptible strains were sequenced. Both resistant and susceptible strains contained a putative macrolide 2′-phosphotransferase gene, but it was noted that all resistant strains contained a gene encoding an ABC-F type ribosomal protection protein Lsa(B) (Additional file 3: Table S1) whose protein protects the ribosome from the antibiotics action. The three resistant strains showed markedly reduced inhibition zone diameters of 10 mm (MS 532a), 14.25 mm (MS 195) and 14.5 mm (MS 735) (Fig. 3a), which were noticeably smaller than the EUCAST suggested breakpoint diameter for *S. aureus* used in this study of < 18 mm. Therefore, this value for erythromycin could be decreased to < 15 mm as a new breakpoint for *Bacillus cereus s.l.* according to the results of this study (Fig. 3a).

The genome of the tetracycline-resistant strain MS 532a, which showed an 8.5 mm diameter zone of inhibition, was sequenced and a gene for a tetracycline resistance efflux pump *tet(*45) was identified that could explain the resistance mechanism (Additional file 3: Table S1). As there are no EUCAST criteria for determining susceptibility of *Bacillus* strains, the EUCAST criteria for *S. aureus* were used in this study. A breakpoint of < 19 mm for tetracycline is specified in this case to indicate resistance, which in our opinion is not suitable for *Bacillus*, as the breakpoints of non-resistant strains ranged from 18 to 33 mm (Fig. 3a). Thus, we would suggest that for *Bacillus cereus s.l.* group strains this value should be < 18 mm or even less.

Strains were also chosen for genome sequencing that appeared to be chloramphenicol or gentamicin resistant. Although the small diameters of the inhibition zones indicated the strains to be clearly resistant, genome sequencing did not detect a specific genetic determinant that could explain the resistance fully. In the case of chloramphenicol, all sequenced strains possessed a chloramphenicol *O*-acetyltransferase of the CatA15/A16 family (Additional file 3: Table S1), yet only one strain (MS532a) was phenotypically resistant (Figs. 2 and 3c). Should this gene be responsible for conferring resistance, the gene must have been present in an inactivated pseudogene form in the sensitive strains. In the case of gentamicin resistance, the strains B26, MS532a and MS464a were phenotypically resistant (Figs. 2 and 3d), yet putative aminoglycoside 6-nucleotidyltransferase genes were detected in all sequenced genomes, of which 3 strains did not show a resistance. Again, this may be possibly a result of the presence of pseudogenes with mutations in susceptible strains or the resistances may rely on other unknown genes and mechanisms.

## Conclusion

The results of our previous study as well as this study show that *B. cereus s.l.* strains can occur in fresh vegetables on the German retail markets at levels of up to ca. log 3.0log 4.0 cfu/g [2, 6] and that these contain different combinations of enterotoxin genes that are known to be responsible for causing diarrhea. The methods for detection of these toxins, i.e. multiplex PCR, genome sequencing and serological toxin detection were shown to lead to somewhat differing results, probably as a result of toxin gene sequence polymorphisms. Thus, a combination of methods should be applied to obtain an accurate assessment of the presence of toxin genes in *B. cereus s.l.* strains. None of the strains were detected to contain the gene for the cereulide toxin. Generally, the levels of fresh vegetable contamination with *B. cereus* were presumably too low to cause toxin production and foodborne disease. This probably explains why the reported incidences of foodborne disease caused by these bacteria after consumption of fresh vegetables in Germany are rather low. However, these bacteria can be consistently isolated from such products. In the case of ready-to-eat products, this shows that there is an inherent risk associated with these products if storage temperatures are abused or if the products become contaminated with higher *B. cereus s.l.* loads. Moreover, the effect of these toxin-producers on vulnerable consumers is not yet known. For these reasons, a close vigil should be kept on the occurrence and levels of *B. cereus s.l.* in such fresh vegetables, especially ready-to-eat products. *Bacillus cereus s.l.* are also generally sensitive towards antibiotics. It could be determined in this study that antibiotic resistance breakpoint values supplied for *S. aureus* strains in EUCAST were suitable for many, but not all antibiotics tested against *B. cereus* group strains. The data generated in this study could be the basis for further studies of such resistance breakpoints. Nevertheless, our results indicate that resistances towards antibiotics such as tetracycline, erythromycin and aminoglycosides, can occur. One strain in this study was multiple resistant towards β-lactam antibiotics, aminoglycoside, tetracycline and erythromycin. The presence of these antibiotic resistance genes in these gram-positive, *Bacillus* microorganisms therefore appears to mirror the current occurrence of such resistance genes in the vegetable environment. Furthermore, it thus represents a possibly less important source of potentially transferable resistance genes in the food chain.

## Methods

### Bacterial strains

A total of 147 *B. cereus s.l.* strains were isolated from 137 fresh vegetable samples, i.e. cucumbers (*n* = 6), carrots (*n* = 11), herbs (*n* = 22), salad leaves (either lollo bionda, lamb’s lettuce, spinach or arugula) (*n* = 19) and ready-to-eat mixed salads (*n* = 89), purchased from retail stores, local markets and farmyard sales in Germany. The isolates and the products from which they were isolated are shown in Table 3. *B. cereus s.l.* strains (*n* = 73) were selectively isolated on *Bacillus-cereus*-Agar (PEMBA; Sifin, Berlin, Germany) at 30 °C for 48 h [2], while 74 further strains were isolated on *Bacillus cereus* Rapid Agar (BACARA; bioMérieux, Nürtingen, Germany) at 30 °C for 48 h (*n* = 74 isolates, see Table 3). Some of the 73 strains isolated on PEMBA agar were previously identified and described [2].

**Table 3:** Origin of *Bacillus cereus*-groupstrains investigated in this study

Source of isolation and no. of samples (137)	Isolates^a^	Total no. of isolates (147)
Carrots (11)	*M5, M6, M10, M14, M15, M18, M19, M20, M26, M27, M39*	11
Cucumbers (6)	*G2, G8, G12, G13, G32, G36*	6
Herbs (22)	*K1, K2, K10, K11, K13, K14, K16, K17, K20, K21, K22, K23, K28, K30, K31, K33, K34, K35, K37, K38, K39, K40*	22
Salad leaves (either one of the following: lollo bionda, lamb’s lettuce, spinach, arugula) (19)	*B2, B8, B15, B16, B19, B20, B24, B25, B26, B27, B29, B30, B31, B32, B34, B36, B37, B38, B40*	19
Mixed salad leaves (79)	*MS3, MS4, MS6, MS9, MS12, MS14, MS17, MS18, MS19, MS25, MS27, MS29, MS33, MS36, MS38,* MS79, MS135a, MS135b, MS136a, MS136b, MS138, MS139, MS142, MS143, MS147, MS190, MS191, MS192, MS194, MS195, MS360a, MS360b, MS361, MS362, MS456, MS457, MS460, MS461, MS463a, MS463b, MS464a, MS464b, MS467, MS468a, MS468b, MS469, MS470, MS471, MS525, MS526a, MS527, MS529, MS530, MS531, MS532a, MS532b, MS567, MS568, MS569, MS575, MS578a, MS578b, MS579, MS581, MS582, MS586, MS588, MS689, MS690, MS691a, MS691b, MS693, MS728a, MS728b, MS729, MS730, MS733, MS734, MS735, MS756, MS758, MS788, MS789, MS790, MS791, MS792, MS793, MS794, MS795	89

^a^ isolates marked in italic type were isolated on PEMBA agar and were previously reported regarding their 16S rRNA gene based identification in the study of Fiedler et al. [2]. All other strains were isolated on BACARA agar

### Phenotypic characterization of *Bacillus cereus-*group isolates

All 147 isolates were tested for growth at different temperatures (4, 7, 10 and 50 °C) for 10 days in Caso or Standard 1 Broth (Merck, Darmstadt, Germany). A hemolysis test was performed by streaking onto commercially prepared Columbia agar (Oxoid, Wesel, Germany) plates containing 7% sheep’s blood and incubating for 24 h at 30 °C. Plates were examined for α-, β- and ɣ-hemolysis.

### Antibiotic susceptibility testing

Antimicrobial susceptibility of the *B. cereus s.l.* species was tested using the disc diffusion method based on the European Committee on Antimicrobial Susceptibility Testing (EUCAST) guidelines [26]. A total of 12 different antibiotic discs (Oxoid) containing the antibiotics amikacin (AMK 30 μg), ampicillin (AMP 10 μg), amoxicillin/clavulanic acid (AMC 20/10 μg), cefotaxime (CTX 30 μg), chloramphenicol (CHL 30 μg), ciprofloxacin (CIP 5 μg), erythromycin (ERY 15 μg), gentamicin (GEN 10 μg), imipenem (IPM 10 μg), penicillin G (PEN 10 μg), tetracycline (TET 30 μg) and trimethoprim-sulfamethoxazole (SXT 25 μg) were used for susceptibility testing on Mueller Hinton Agar (Merck). Susceptibility of the isolates to the antibiotics was determined by measuring the zone of inhibition, and these were interpreted as sensitive, intermediate or resistant according to EUCAST [26] criteria for *Staphylococcus* (*S*.) *aureus* (except imipenem and amoxicillin/clavulanic acid), as no *Bacillus*-specific criteria for disc diffusion antibiotic-resistance assays have been defined either by EUCAST or Clinical & Laboratory Standards Institute guidelines (CSLI). For imipenem, the *Enterococcus* spp. (EUCAST) and for amoxicillin/clavulanic acid, the *Enterobacteriales* (EUCAST) breakpoints were adopted. All antibiotic resistance determinations were carried out in duplicate on different days, using different agar batches and different overnight-subcultures. For ambiguous results (difference between duplicates > 4 mm) a third repetition was performed. Data were graphically presented using JMP (v14, SAS software, Cary, USA).

### Duopath® test for detection of Nhe and Hbl bacterial toxin formation

The Duopath® Cereus Enterotoxins test (Merck) test detects the NheB component of the non-haemolytic three-component enterotoxin (Nhe) and the HblC component of the three-component haemolysin BL (Hbl) [40] and was performed using overnight Bacillus isolates broth cultures. The *Bacillus* isolates were streaked onto mannitol egg yolk polymyxin (MYP) agar (Oxoid). After incubation at 30 °C for 18 to 24 h, one to three colonies were picked and suspended in 1 ml of casein hydrolysate-glucose-yeast extract broth (CGY) (Merck). After 4 h incubation at 37 °C, a 150 μl aliquot of the enrichment was used to inoculate the Duopath® Cereus Enterotoxins lateral flow immunoassay and incubated for 30 min at room temperature. The test was considered positive if a red line became visible within 30 min in both the test (Nhe and /or Hbl) and control zones and negative if a line appeared only in the control zone.

### DNA extraction and 16S rRNA gene sequencing

The cultures were grown overnight in Caso Broth or Standard 1 Broth, and DNA was extracted either using the ZR Fungal/Bacterial DNA MiniPrep™ (Zymo Research, Freiburg, Germany) according to the manufacturer’s instructions or alternatively according to Pitcher et al. (1989). DNA concentration was quantified using NanoDrop 2000 (Thermo Scientific, Waltham, USA) or using a Qubit fluorometer (Invitrogen, Darmstadt, Germany).

For genotypic characterization, the 86% complete 16S rRNA gene sequence of the isolates (ca. 1342 bp) was obtained as previously described by Fiedler et al. [2] and Danylec et al. [41]. The sequences were compared and phylogenetic analyses were performed by fast algorithm and unweighted pair group method with arithmetic mean (UPGMA) clustering using BioNumerics (v7.6, Applied Maths, Saint-Martens-Latem, Belgium).

### PCR screening for enterotoxin genes

*Bacillus cereus s.l.* strains toxin genes were detected by multiplex PCR according to Ehling-Schulz et al. [16]. This multiplex PCR was designed to simultaneously amplify the enterotoxin genes *hblDA*, *nheAB, cytK-2* and *ces* with PCR product sizes of 1091 bp, 766 bp, 421 bp and 1271 bp, respectively. Suitable reference strains from the DSMZ culture collection were used as positive controls. The multiplex PCR reactions were done independently at two different laboratories at different departments of the Max Rubner-Institut, i.e. the Department of Safety and Quality of Fruit and Vegetables located in Karlsruhe and the Department of Microbiology and Biotechnology located in Kiel, using the method of Ehling-Schulz et al. [16] with slight variations (see Additional file 4: Table S2). The PCR products were separated by gel electrophoresis, stained with ethidium bromide (Merck) and investigated using a UV transilluminator (VWR).

### Whole genome sequencing and analyses

The sequencing library was prepared with an Illumina Nextera XT library prep kit (Illumina, San Diego, USA) and sequenced on a MiSeq (Illumina) sequencer with 2 × 250 paired-end reads. The reads were de novo assembled using SPAdes version 3.11.1 [42]. The genome sequence was annotated and assigned to *B. cereus* multi locus sequence types using the PATRIC database [43]. The acquired antibiotic resistance genes were identified using the ResFinder server (v. 3.0) [44]. Genome and protein sequences were analyzed and comparisons were visualized using Geneious (v9.0.5, Biomatters Limited, New Zealand). In order to construct a phylogenetic tree based on genome sequences, the annotated amino acid sequence encoded by each genome were extracted and utilized by the PATRIC server. A homologous group filtering and a group alignment were performed by this pipeline and an estimated phylogenetic tree from concatenated alignment sequences was calculated with the outgroup type strain *Geobacillus thermoglucosidasius* DSM 2542^T^ using a FastTree method [25].

## Supplementary information

**Additional file 1: Figure S1.** Cluster analysis of 16S rRNA gene sequences of 147 *Bacillus cereus s.l.* and selected type strains (*B. anthracis* ATCC 14578^T^, *B. cereus* DSM 31^T^, *B. cytotoxicus* DSM 22905^T^, *B. mycoides* DSM 2048^T^, *B. pseudomycoides* DSM 12442^T^, *B. subtilis* DSM 10^T^, *B. thuringiensis* DSM 2046^T^, *B. toyonensis* CECT 876^T^, *B. weihenstephanensis* DSM 11821^T^). Strains isolated in Kiel and Karlsruhe are labelled green and red, respectively. Reference strains are labelled in blue. Fast algorithm as similarity coefficient and UPGMA were used. Due to the high number of strains the dendrogram was condensed.

**Additional file 2: Figure S2.** (a) Primer mismatches *nheA*: Analysis of the *nheA* region targeted by the PCR Primer NA2F. The yellow box indicates a nucleotide mismatch and the nucleotide positions are indicated above the primer sequence. Inosine was used as a degenerate base and was marked with an I in the primer sequence. (b) Primer mismatches *hblD*: Analysis of the *hblD* region targeted by PCR with Primer HD2F. A yellow box indicates a nucleotide mismatch and the nucleotide positions are indicated above the primer sequence. Inosine was used as a degenerate base and was marked with an I in the primer sequence.

**Additional file 3: Table S1.** Antibiotic resistance genes identified on the genomes of *B. cereus*-group strains using the PATRIC [43] database (bold highlights acquired resistance genes identified by ResFinder [45]).

**Additional file 4: Table S2.** Multiplex PCR conditions used for toxin gene amplification.

## Data Availability

The data-sets analyzed during the current study are available from the corresponding author on reasonable request. The *Bacillus cereus s.l.* genome sequences (B26, G12, MS12, MS17, MS195, MS464a, MS532a and MS735) are available on the NCBI Genome Database under the accession numbers SJQA00000000, SJQB00000000, SJQC00000000, SJQD00000000, SJQE00000000, SJQF00000000, SJQG00000000 and SJQH00000000, respectively.

## Abbreviations

ATCC: American Type Culture Collection
*B.*: *Bacillus*
bp: Base pair
*ces*: cereulide
CLSI: Clinical and Laboratory Standards Institute guidelines
*cytK*: cytotoxin K
DNA: deoxyribonucleic acid
DSMZ: Deutsche Sammlung von Mikroorganismen und Zellkulturen
EUCAST: European Committee on Antimicrobial Susceptibility Testing
*hbl*: hemolysin BL
mbp: mega base pair
MLST: multilocus sequence typing
mm: millimeter
*nhe*: non-hemolytic enterotoxin
PCR: polymerase chain reaction
PEMBA: Polymyxin pyruvate egg yolk mannitol bromothymol blue agar
rRNA: ribosomal ribonucleic acid
*S.*: *Staphylococcus*
*s*.*l*.: sensu *lato*
*s*.*s*.: sensu stricto
UPGMA: unweighted pair group method (with) arithmetic mean
