# Supplementary figures and images for: Antibiotics resistance and toxin profiles of Bacillus cereus-group isolates from fresh vegetables from German retail markets
Source: BMC Microbiol. 2019 Nov 9;19:250. doi: 10.1186/s12866-019-1632-2 (PMC6842220; doi:10.1186/s12866-019-1632-2)

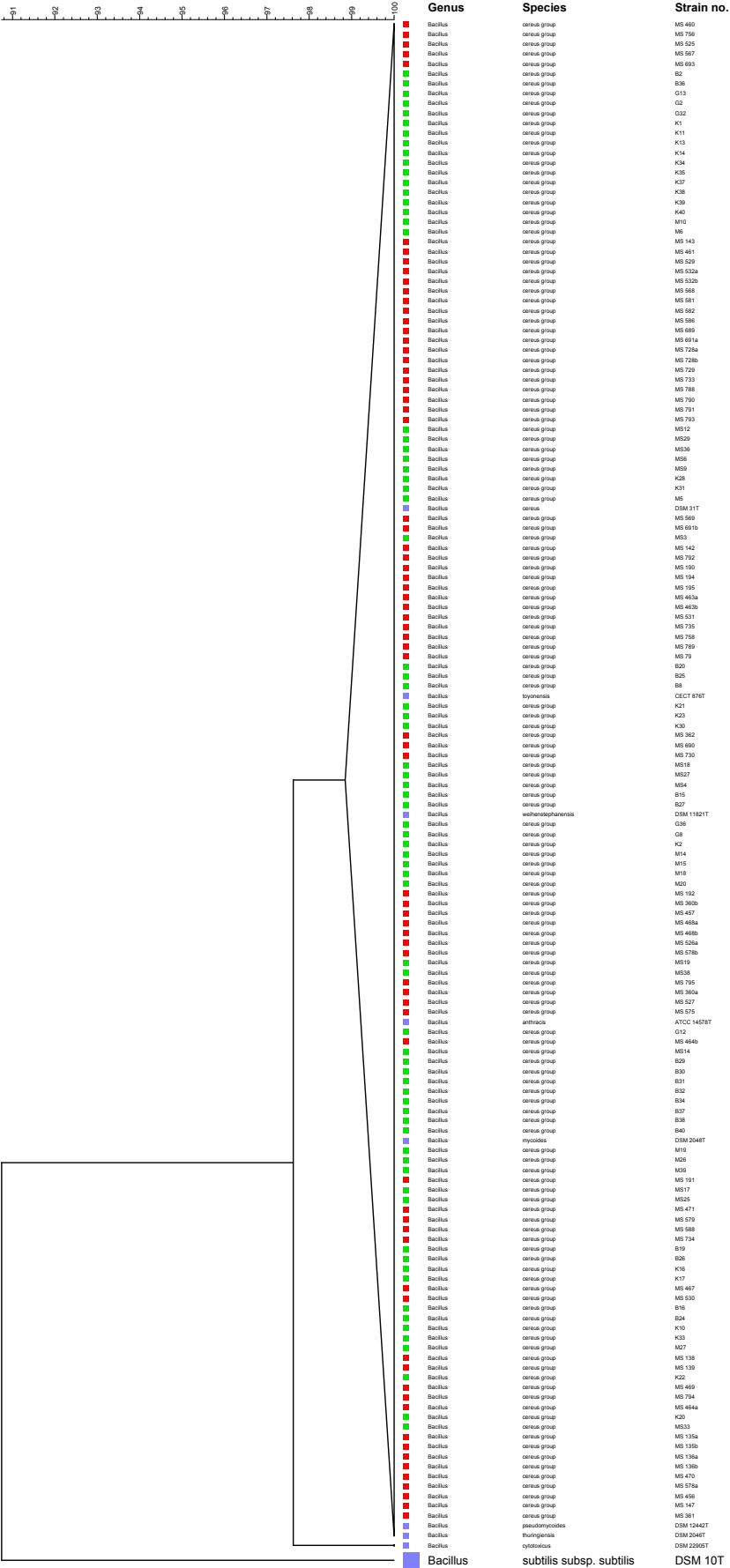

Supplement: Supplementary file 1 — Additional file 1: Figure S1. Cluster analysis of 16S rRNA gene sequences of 147 Bacillus cereus s.l. and selected type strains (B. anthracis ATCC 14578T, B. cereus DSM 31T, B. cytotoxicus DSM 22905T, B. mycoides DSM 2048T, B. pseudomycoides DSM 12442T, B. subtilis DSM 10T, B. thuringiensis DSM 2046T, B. toyonensis CECT 876T, B. weihenstephanensis DSM 11821T). Strains isolated in Kiel and Karlsruhe are labelled green and red, respectively. Reference strains are labelled in blue. Fast algorithm as similarity coefficient and UPGMA were used. Due to the high number of strains the dendrogram was condensed. [file 12866_2019_1632_MOESM1_ESM.pdf]
