# Supplementary material for: Antibiotics resistance and toxin profiles of Bacillus cereus-group isolates from fresh vegetables from German retail markets
Source: BMC Microbiol. 2019 Nov 9;19:250. doi: 10.1186/s12866-019-1632-2 (PMC6842220; doi:10.1186/s12866-019-1632-2)
Supplement: Supplementary file 2 — Additional file 2: Figure S2. (a) Primer mismatches nheA: Analysis of the nheA region targeted by the PCR Primer NA2F. The yellow box indicates a nucleotide mismatch and the nucleotide positions are indicated above the primer sequence. Inosine was used as a degenerate base and was marked with an I in the primer sequence. (b) Primer mismatches hblD: Analysis of the hblD region targeted by PCR with Primer HD2F. A yellow box indicates a nucleotide mismatch and the nucleotide positions are indicated above the primer sequence. Inosine was used as a degenerate base and was marked with an I in the primer sequence. [file 12866_2019_1632_MOESM2_ESM.pdf]

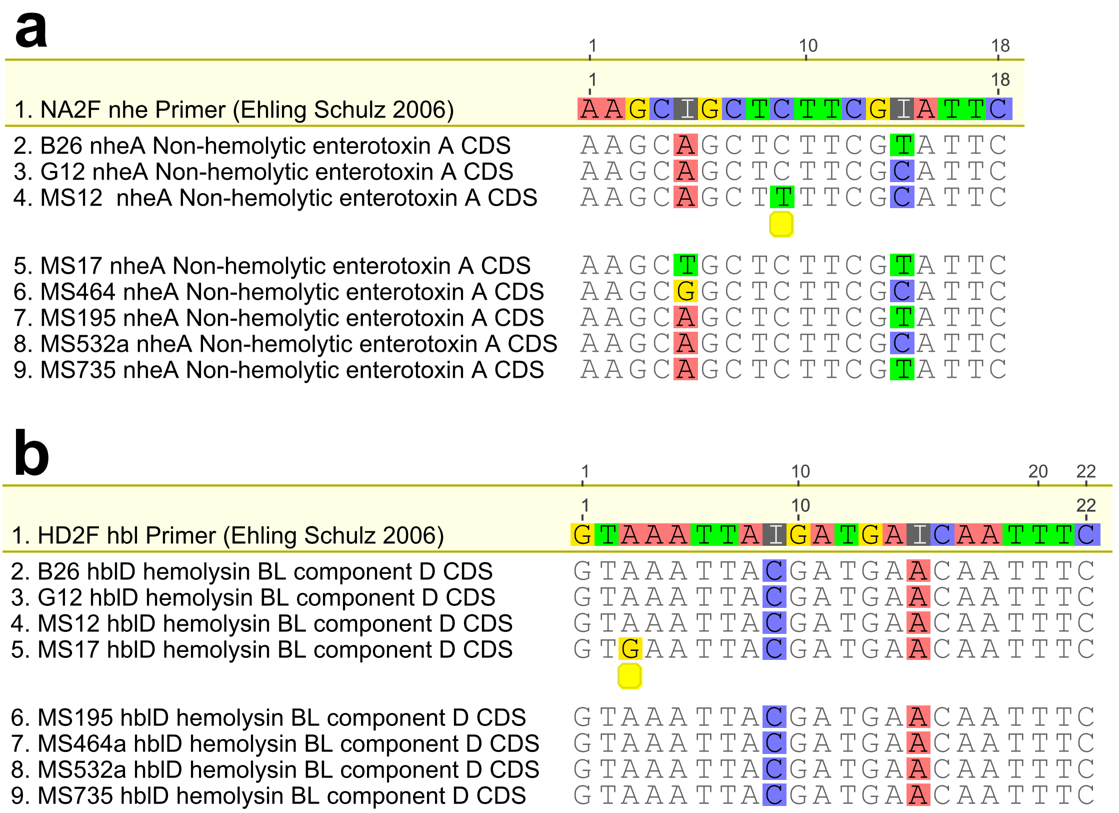
a
1
10
18
1. NA2F nhe Primer (Ehling Schulz 2006)
AAGCIGCTCTTCGIATTC
2. B26 nheA Non-hemolytic enterotoxin A CDS
AAGCAGCTCTTCGTATTC
3. G12 nheA Non-hemolytic enterotoxin A CDS
AAGCAGCTCTTCGCATTC
4. MS12 nheA Non-hemolytic enterotoxin A CDS
AAGCAGCTTTTCGCATTC
5. MS17 nheA Non-hemolytic enterotoxin A CDS
AAGCTGCTCTTCGTATTC
6. MS464 nheA Non-hemolytic enterotoxin A CDS
AAGCGGCTCTTCGCATTC
7. MS195 nheA Non-hemolytic enterotoxin A CDS
AAGCAGCTCTTCGTATTC
8. MS532a nheA Non-hemolytic enterotoxin A CDS
AAGCAGCTCTTCGCATTC
9. MS735 nheA Non-hemolytic enterotoxin A CDS
AAGCAGCTCTTCGTATTC
b
1
10
20
22
1. HD2F hbl Primer (Ehling Schulz 2006)
GTAAATTAIGATGAICAATTTC
2. B26 hblD hemolysin BL component D CDS
GTAAATTACGATGAACAATTTC
3. G12 hblD hemolysin BL component D CDS
GTAAATTACGATGAACAATTTC
4. MS12 hblD hemolysin BL component D CDS
GTAAATTACGATGAACAATTTC
5. MS17 hblD hemolysin BL component D CDS
GTGAATTACGATGAACAATTTC
6. MS195 hblD hemolysin BL component D CDS
GTAAATTACGATGAACAATTTC
7. MS464a hblD hemolysin BL component D CDS
GTAAATTACGATGAACAATTTC
8. MS532a hblD hemolysin BL component D CDS
GTAAATTACGATGAACAATTTC
9. MS735 hblD hemolysin BL component D CDS
GTAAATTACGATGAACAATTTC
