# Supplementary material for: Antibiotics resistance and toxin profiles of Bacillus cereus-group isolates from fresh vegetables from German retail markets
Source: BMC Microbiol. 2019 Nov 9;19:250. doi: 10.1186/s12866-019-1632-2 (PMC6842220; doi:10.1186/s12866-019-1632-2)
Supplement: Supplementary file 3 — Additional file 3: Table S1. Antibiotic resistance genes identified on the genomes of B. cereus-group strains using the PATRIC [43] database (bold highlights acquired resistance genes identified by ResFinder [45]). [file 12866_2019_1632_MOESM3_ESM.docx]

**Additional file 3: Table S1** Antibiotic resistance genes identified on the genomes of *B. cereus* group strains using the PATRIC [28] database (bold highlights acquired resistance genes identified by ResFinder [46]).

| **Strain**  **(antibiotic resistance phenotype)** | **Putative resistance genes identified on genome (PATRIC database)** | | | | | |
| --- | --- | --- | --- | --- | --- | --- |
|  | Erythromycin | Chloramphenicol | Aminoglycoside | Tetracycline | Beta Lactams | Others |
| **B26**  phenotypic resistance to AP, CTX, AUG, PG, TS, GM |  |  | Aminoglycoside 6-nucleotidyltransferase, putative |  | Class A beta-lactamase (EC 3.5.2.6) | Fosfomycin resistance protein FosB |
|  |  |  | weak similarity to aminoglycoside N(3)-acetyltransferase |  | Subclass B1 beta-lactamase (EC 3.5.2.6) => BcII family β-lactamases | Streptogramin O-acetyltransferase, putative |
|  |  |  |  |  |  | Broad-specificity multidrug efflux pump YkkC |
| **G12**  phenotypic resistance to AP, CTX, AUG, PG, TS | Macrolide 2'-phosphotransferase, putative | Chloramphenicol O-acetyltransferase (EC 2.3.1.28) => CatA15/A16 family | Aminoglycoside 6-nucleotidyltransferase, putative |  | Class A beta-lactamase (EC 3.5.2.6) | Fosfomycin resistance protein FosB |
|  | ABC-F type ribosomal protection protein => Lsa(B) |  | weak similarity to aminoglycoside N(3)-acetyltransferase |  | Subclass B1 beta-lactamase (EC 3.5.2.6) => BcII family β-lactamases | Streptogramin O-acetyltransferase, putative |
|  |  |  |  |  |  | Broad-specificity multidrug efflux pump YkkC |
| **MS12**  phenotypic resistance to AP, CTX, AUG, PG | Macrolide 2'-phosphotransferase, putative | Chloramphenicol O-acetyltransferase (EC 2.3.1.28) => CatA15/A16 family | Aminoglycoside 6-nucleotidyltransferase, putative |  | Class A beta-lactamase (EC 3.5.2.6) | Fosfomycin resistance protein FosB |
|  | ABC-F type ribosomal protection protein => Lsa(B) |  | weak similarity to aminoglycoside N(3)-acetyltransferase |  | Subclass B1 beta-lactamase (EC 3.5.2.6) => BcII family β-lactamases | Streptogramin O-acetyltransferase, putative |
|  |  |  |  |  |  | Broad-specificity multidrug efflux pump YkkC |
| **MS17**  phenotypic resistance to AP, CTX, AUG, PG, TS |  |  | Aminoglycoside 6-nucleotidyltransferase, putative |  | Class A beta-lactamase (EC 3.5.2.6) | Fosfomycin resistance protein FosB |
|  |  |  | weak similarity to aminoglycoside N(3)-acetyltransferase |  | Subclass B1 beta-lactamase (EC 3.5.2.6) => BcII family β-lactamases | Streptogramin O-acetyltransferase, putative |
|  |  |  |  |  |  | Broad-specificity multidrug efflux pump YkkC |
|  |  |  |  |  |  | Bacitracin export ATP-binding protein BceA |
| **MS195**  phenotypic resistance to AP, CTX, AUG, PG, E | Macrolide 2'-phosphotransferase, putative | Chloramphenicol O-acetyltransferase (EC 2.3.1.28) => CatA15/A16 family | Aminoglycoside 6-nucleotidyltransferase, putative |  | Class A beta-lactamase (EC 3.5.2.6) | Fosfomycin resistance protein FosB |
|  | ABC-F type ribosomal protection protein => Lsa(B) |  | weak similarity to aminoglycoside N(3)-acetyltransferase |  | Subclass B1 beta-lactamase (EC 3.5.2.6) => BcII family β-lactamases | Streptogramin O-acetyltransferase, putative |
|  |  |  |  |  |  | Broad-specificity multidrug efflux pump YkkC |
| **MS464a**  phenotypic resistance to AP, CTX, AUG, PG, TS, GM | Macrolide 2'-phosphotransferase, putative | Chloramphenicol O-acetyltransferase (EC 2.3.1.28) => CatA15/A16 family | Aminoglycoside 6-nucleotidyltransferase, putative |  | Class A beta-lactamase (EC 3.5.2.6) | Fosfomycin resistance protein FosB |
|  |  |  | weak similarity to aminoglycoside N(3)-acetyltransferase |  | Subclass B1 beta-lactamase (EC 3.5.2.6) => BcII family β-lactamases | Streptogramin O-acetyltransferase, putative |
|  |  |  |  |  |  | Broad-specificity multidrug efflux pump YkkC |
|  |  |  |  |  |  | **Vancomycin response regulator VanR** |
| **MS532a**  phenotypic resistance to AP, CTX, AUG, PG, T, GM, E, C | Macrolide 2'-phosphotransferase, putative | Chloramphenicol O-acetyltransferase (EC 2.3.1.28) => CatA15/A16 family | Aminoglycoside 6-nucleotidyltransferase, putative | **Tetracycline resistance, MFS efflux pump => Tet(45)** | Class A beta-lactamase (EC 3.5.2.6) | Fosfomycin resistance protein FosB |
|  | ABC-F type ribosomal protection protein => Lsa(B) |  | weak similarity to aminoglycoside N(3)-acetyltransferase |  | Subclass B1 beta-lactamase (EC 3.5.2.6) => BcII family β-lactamases | Streptogramin O-acetyltransferase, putative |
|  |  |  |  |  |  | Broad-specificity multidrug efflux pump YkkC |
|  |  |  |  |  |  | **Vancomycin response regulator VanR** |
| **MS735**  phenotypic resistance to AP, CTX, AUG, PG, E | Macrolide 2'-phosphotransferase, putative | Chloramphenicol O-acetyltransferase (EC 2.3.1.28) => CatA15/A16 family | Aminoglycoside 6-nucleotidyltransferase, putative |  | Class A beta-lactamase (EC 3.5.2.6) | Fosfomycin resistance protein FosB |
|  | ABC-F type ribosomal protection protein => Lsa(B) |  | weak similarity to aminoglycoside N(3)-acetyltransferase |  | Subclass B1 beta-lactamase (EC 3.5.2.6) => BcII family β-lactamases | Streptogramin O-acetyltransferase, putative |
|  |  |  |  |  |  | Broad-specificity multidrug efflux pump YkkC |
