# Supplementary material for: Antibiotics resistance and toxin profiles of Bacillus cereus-group isolates from fresh vegetables from German retail markets
Source: BMC Microbiol. 2019 Nov 9;19:250. doi: 10.1186/s12866-019-1632-2 (PMC6842220; doi:10.1186/s12866-019-1632-2)
Supplement: Supplementary file 4 — Additional file 4: Table S2. Multiplex PCR conditions used for toxin gene amplification. [file 12866_2019_1632_MOESM4_ESM.docx]

**Additional file 4: Table S2** Multiplex PCR conditions used for toxin gene amplification.

| **Primers** | **Toxin gene** | **Composition acc. to Ehling Schulz et al. [1]** | **Composition Department 1 (Kiel)** | **Composition Department 2 (Karlsruhe)** |
| --- | --- | --- | --- | --- |
| HD2F | *hbl*; hemolysin BL | 1.0 µM | 1.0 µM | 0.25 µM |
| HA4R |  |  |  |  |
| NA2F | *nhe*; non-hemolytic enterotoxin | 0.3 µM | 0.3 µM | 0.075 µM |
| NB1R |  |  |  |  |
| CKF2 cytK | *cytK-2*; cytotoxin K | 0.4 µM | 0.4 µM | 0.1 µM |
| CKR5 cytK |  |  |  |  |
| CesF1 | *ces*; cereulid | 0.2 µM | 0.2 µM | 0.05 µM |
| CesR2 |  |  |  |  |
|  | | Concentration acc. to Ehling Schulz et al. [1] | Concentration Department 1 (Kiel) | Concentration Department 2 (Karlsruhe) |
| Mastermix | | 1U of ThermoStart Taq DNA Polymerase | 1x DreamTaq™ PCR Master Mix | 1x ALLin^TM^ Hot Start Taq Mastermix |
| Final concentration MgCl_2_ | | 3 mM | 2.5 mM | 3 mM |
| Genomic DNA template | | 1 µl of unspecified concentration | 5 µl (10 ng/µl) | 10 µl (10 ng/µl) |
| PCR cycler and program | | n.a.  95 °C for 15 min,  30 cycles  [95 °C for 30 s,  49 °C for 30 s,  72 °C for 60 s]  72 °C for 2 min | Biorad C1000  95 °C for 3 min,  33 cycles  [95 °C for 30 s,  49 °C for 30 s,  72 °C for 60 s]  72 °C for 5 min | peQStar  95 °C for 5 min,  30 cycles  [95 °C for 30 s,  49 °C for 30 s,  72 °C for 60 s]  72 °C for 2 min |
